# Identification of four plasma microRNAs as potential biomarkers in the diagnosis of male lung squamous cell carcinoma patients in China

**DOI:** 10.1002/cam4.1490

**Published:** 2018-04-19

**Authors:** Xia Shan, Huo Zhang, Lan Zhang, Xin Zhou, Tongshan Wang, JinYing Zhang, Yongqian Shu, Wei Zhu, Wei Wen, Ping Liu

**Affiliations:** ^1^ Department of Oncology First Affiliated Hospital of Nanjing Medical University 300 Guangzhou Road Nanjing 210029 China; ^2^ Department of Respiration The Affiliated Jiangning Hospital of Nanjing Medical University Nanjing 210000 China; ^3^ Department of Radiation Oncology Suzhou Municipal Hospital Suzhou Cancer Center The Affiliated Suzhou Hospital of Nanjing Medical University Suzhou Jiangsu 215001 China; ^4^ Department of Oncology The Affiliated Jiangsu Shengze Hospital of Nanjing Medical University No.1399 West Road Wujiang District Suzhou 215000 China; ^5^ Department of Thoracic Surgery First Affiliated Hospital of Nanjing Medical University 300 Guangzhou Road Nanjing 210029 China

**Keywords:** Diagnostic biomarker, lung squamous cell carcinoma, microRNA, plasma, qRT‐PCR

## Abstract

Dysregulated microRNAs (miRNAs) in the plasma of patients with lung squamous cell carcinoma (LSCC) might serve as biomarkers for LSCC diagnosis. The expression of miRNAs was performed using quantitative reverse transcription–polymerase chain reaction (qRT‐PCR) on the basis of Exiqon panels in the initial screening phase including three male LSCC pool samples and one normal control (NC) pool sample (per 10 samples were pooled as one pool sample). After the training (32 LSCC vs. 31 NCs), the testing (55 LSCC vs. 55 NCs), and the external validation (15 LSCC vs. 15 NCs) stages via qRT‐PCR, a four‐miRNA signature (miR‐181a‐5p, miR‐21‐5p, miR‐106a‐5p, and miR‐93‐5p) was identified for LSCC detection. Areas under the receiver operating characteristic (ROC) curve (AUC) of the four‐miRNA panel for the training, the testing, and the external validation phases were 0.795, 0.827, and 0.914, respectively. Then, the four miRNAs were explored in LSCC tissue samples (23 LSCC vs. 23 NCs), and their expression was significantly up‐regulated. However, none of the four miRNAs found significantly up‐regulated in plasma exosomes expect miR‐93‐5p with borderline significance (16 LSCC vs. 16 NCs). In summary, our study established a four‐miRNA peripheral plasma signature, which contributed to diagnosing male LSCC patients in China to a certain degree.

## Introduction

With increasing incidence and mortality, lung cancer has become one of the most fatal malignancies internationally 1. Non‐small‐cell lung cancer (NSCLC) consist of approximately 85 percent among all types of lung cancer, and are further classified into lung squamous cell carcinoma (LSCC) and adenocarcinoma (AC) 2. Male patients with smoking history occupy the majority of LSCC 3. Recently, treatment of NSCLC has entered into the era of “personalized medicine,” especially for patients with molecular drivers. Unfortunately, few breakthroughs have been made in the treatment of LSCC, and five‐year survival rate for advanced LSCC proved still less than 15% 2. However, high mortality can be significantly decreased if early‐stage LSCC is detected. Low‐dose computed tomography (LDCT) is recommended as one of the screening methods highly applied for LSCC diagnosis 4. However, clinical application of LDCT is limited due to potential harmful effects induced by radiation and overdiagnosis. Some noninvasive biomarkers—neuron‐specific enolase (NSE), Cyfra 211—and squamous cell carcinoma antigen (SCC Ag), for example, are not specific and sensitive enough to facilitate early detection of LSCC 5. Promisingly, with the development of new technologies, noninvasive and novel diagnostic markers with high efficiency are being discovered for the detection of early LSCC.

MicroRNAs (miRNAs), a length of 19~22 nucleotides, are a series of endogenous noncoding RNAs. They function upon regulating gene expression and RNA silencing in post‐transcriptional level 6. Previous researches indicate that deregulation of miRNAs is involved in initiation and progression of various cancers 7. Furthermore, circulating miRNAs are stably preserved and reducibly measurable in blood and can serve as reliable biomarkers for the diagnosis and prognosis for NSCLC 8, 9, 10, 11, 12. However, the results so far were inconsistent due to different research methods or heterogeneous histological subtypes. Analysis of circulating tumor‐derived miRNAs provides a potential approach for the diagnosis of lung cancer and its different subtypes. For example, based on quantitative reverse transcription–polymerase chain reaction (qRT‐PCR), we have developed a panel of six plasma miRNA biomarkers, including miR‐19b‐3p, miR‐21‐5p, miR‐221‐3p, miR‐409‐3p, miR‐425‐5p, and miR‐584‐5p for the specific diagnosis of AC 13. Furthermore, we have previously identified a three‐miRNA peripheral serum signature consisting of miR‐93‐5p, miR‐106a‐5p and miR‐20a‐5p for the detection of male patients with LSCC from normal controls (NCs) 14. Thus, we expect to identify a specific plasma miRNA expression profile that can further assist the detection of LSCC in Chinese male patients.

## Methods

### Study population

Written informed consent was obtained from each participant in our study. The male patients enrolled were recruited from Jiangsu Province People's Hospital of Nanjing Medical University during 2012 and 2015 and histopathologically confirmed as LSCC. Peripheral blood samples were drawn before any therapeutic procedures, such as surgery, preoperative radiation, and chemotherapy. According to the seventh edition American Joint Committee on Cancer (AJCC), information of TNM stage and differentiation degree was obtained from patients’ records, retrospectively. Age‐ and sex‐matched normal controls were obtained from healthy individuals taking a routine physical examination at Jiangsu Province People's Hospital of Nanjing Medical University. The study was approved by institutional review board and the Ethics Committee of Nanjing Medical University. All participants provided informed consent.

### Study design

**Figure 1:**
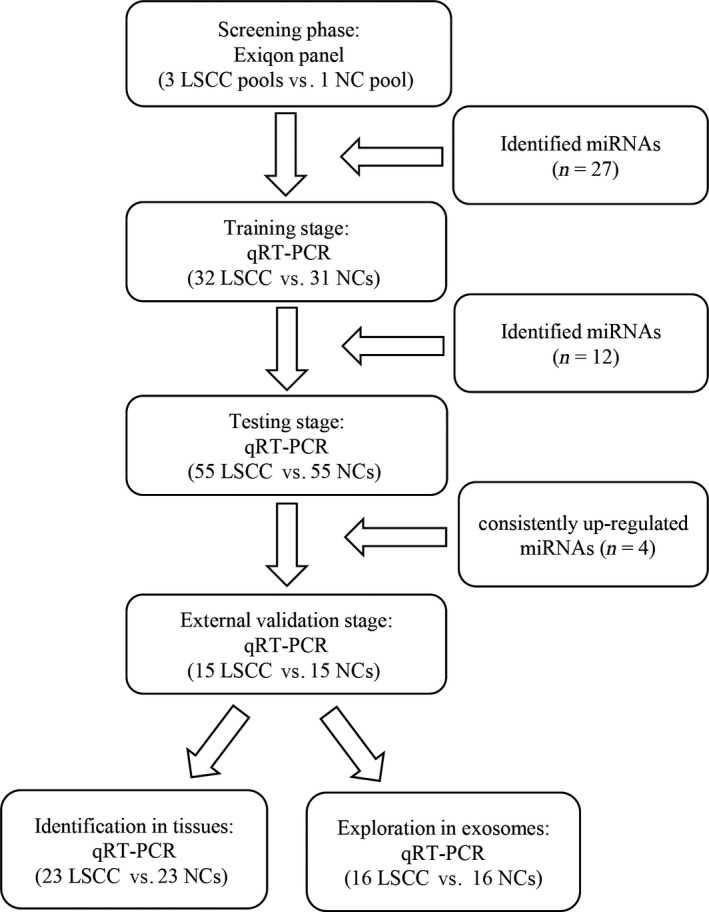
The flowchart of the experiment design. LSCC, lung squamous cell carcinoma; NC, normal control.

A total of 102 LSCC patients and 101 healthy controls were enrolled in our study. The experimental process was designed into four stages (Fig. 1). In the initial screening stage, thirty peripheral plasma samples from LSCC patients and 10 from NCs were randomly selected and pooled as three LSCC samples and one NC sample (10 samples were pooled as one pool sample) for miRNA microarrays via Exiqon‐miRCURY‐Ready‐to‐Use‐PCR‐Human‐panel‐I+II‐V1.M (Exiqon miRNA qPCR panel, Vedbaek, Denmark). Then, in order to verify reproducibility and reliability of the results which acquired from arrays, plasma samples from 32 LSCC samples and 31 NC samples were used to confirm the dysregulated miRNAs in training stage. In following testing stage, the selected miRNAs were refined from 55 LSCC samples and 55 NC samples. In external validation stage, 15 LSCC samples and 15 NC samples were enrolled to further assess the diagnostic performance of the selected miRNAs. These identified miRNAs were validated by 23 pairs of formalin‐fixed paraffin‐embedded (FFPE) LSCC tissue and matched adjacent normal tissue specimens from the same surgery patients. The potential form of miRNAs in the peripheral circulation was investigated via testing the expression of plasma exosomal miRNAs in 16 LSCC samples and 16 NC samples.

Venous blood samples of NC and LSCC samples were collected and placed in an ethylenediaminetetraacetic acid (EDTA)‐containing tube (Becton, Dickinson and Company). Peripheral plasma was isolated from whole blood within 12 hours after collection and then stored at −80°C for further analysis. Tissue specimens were stored in liquid nitrogen.

### Exosomes isolation

In accordance with manufacturer's protocol, exosomes from peripheral plasmas were isolated using ExoQuick^™^ (System Biosciences, Mountain View, CA). Briefly, 200 *μ*L plasma was mixed with 50 *μ*L ExoQuick exosome precipitation solution; exosome pellet was lysed in 200 *μ*L RNAse‐free water for further use.

### RNA extraction

Total RNA was extracted from 200 *μ*L plasma and exosomes solution via the mirVana PARIS Kit (Ambion, Austin, TX). Denaturing solution (Ambion) and 5 *μ*L of synthetic *C. elegans miR‐39* for normalization (5nM/L, RiboBio, China) were added in proper sequence. Tissue RNA was extracted via TRIzol (Invitrogen, Carlsbad, CA). Finally, RNA was dissolved in 100 *μ*L RNAse‐free water. These RNA samples were stored at −40°C until use. Concentration and purification of RNA were evaluated by ultraviolet spectrophotometer.

### Quantitative real‐time polymerase chain reaction (qRT‐PCR)

The amplification of miRNA was performed using specific primers of reverse transcription (RT) and polymerase chain reaction (PCR) (RiboBio, China). The qRT‐PCR was run on a LightCycler^®^ 480 (Roche 480, Germany) real‐time thermal cycler to amplify and detect the miRNAs with SYBR Green dye. Plasma and exosomal miRNAs were calculated using the 2^−ΔΔCt^ method relative to exogenous reference miRNA (*cel‐miR‐39*), ΔCt = Ct*miRNA* − Ct*cel‐miR‐39*. Tissue miRNAs were determined by 2^−ΔΔCt^ method normalized to reference *RNU6B (U6)*.

### Statistical analysis

The different expression levels of miRNAs between LSCC and NC samples were analyzed by Mann–Whitney test. The relationship between miRNAs and clinical features was evaluated using one‐way ANOVA or χ^2^ test. Receiver operating characteristic (ROC) curves and area under the ROC curve (AUC) were used to assess the diagnostic performance of the candidate miRNAs for LSCC. Statistical analyses were performed with SPSS (version 19.0, IBM, Armonk, New York, USA). Differences were considered significant when *P *<* *0.05.

## Results

### Patient description

In total, 203 participants including 102 LSCC patients and 101 NCs were enrolled in our study. Plasma samples were randomly divided into three independent phases that included: the training set, the testing set, and the external validation set (Fig. 1). Demographics and their clinical parameters of LSCC samples and NC samples are listed in Table 1. No significant differences were found in the distribution of gender, age, and smoking history between the LSCC and NC samples.

**Table 1:** Clinical characteristics of 102 LSCC patients and 101 normal controls

Variables	Screening phase (*n* = 40)		*P*	Training stage (*n* = 63)		*P*	Testing stage (*n* = 110)		*P*	External validation stage (*n* = 30)		*P*
	Cases (%)	Controls (%)		Cases (%)	Controls (%)		Cases (%)	Controls (%)		Cases (%)	Controls (%)	
Number	30	10		32	31		55	55		15	15	
Age												
<65	12 (40.0)	3 (30)	>0.05	15 (46.9)	20 (64.5)	>0.05	29 (52.7)	22 (40.0)	>0.05	9 (60.0)	8 (53.3)	>0.05
≥65	18 (60.0)	7 (70)		17 (53.1)	11 (35.5)		26 (47.3)	31 (56.4)		6 (40.0)	7 (46.7)	
Smoking history												
No	4 (13.3)	1 (10.0)	>0.05	3 (9.4)	6 (19.4)	>0.05	6 (10.9)	8 (14.5)	>0.05	1 (6.7)	3 (20.0)	>0.05
Yes	26 (86.7)	9 (90.0)		29 (90.6)	25 (80.6)		49 (89.1)	47 (85.5)		14 (93.3)	12 (80.0)	
TNM stage												
I	9 (30.0)			7 (21.9)			16 (29.1)			3 (20.0)		
II	11 (36.7)			17 (53.1)			26 (47.3)			7 (46.7)		
III	10 (33.3)			8 (25.0)			13 (23.6)			5 (33.3)		
Differentiation												
Well	2 (6.7)			6 (18.8)			11 (20.0)			4 (26.7)		
Moderately	21 (70.0)			19 (59.4)			25 (45.5)			8 (53.3)		
Poorly	7 (23.3)			7 (21.8)			19 (34.5)			3 (20.0)		

### Candidate miRNAs discovered from plasma sample pools

We initially analyzed the expression level of 168 miRNAs to identify candidate miRNAs by Exiqon miRNA qPCR panel in three LSCC and one NC sample pools.

miRNAs in accordance with the following criteria were selected for further analysis: (1) with five Ct lower (or higher) than NCs; (2) exhibiting a cycle threshold (Ct) value <37; (3) at least a 1.5‐fold altered expression. Based on this standard, 19 up‐regulated miRNAs and eight down‐regulated miRNAs were selected for further assessment (Table S1).

### Confirmation of identified miRNAs by qRT‐PCR

**Figure 2:**
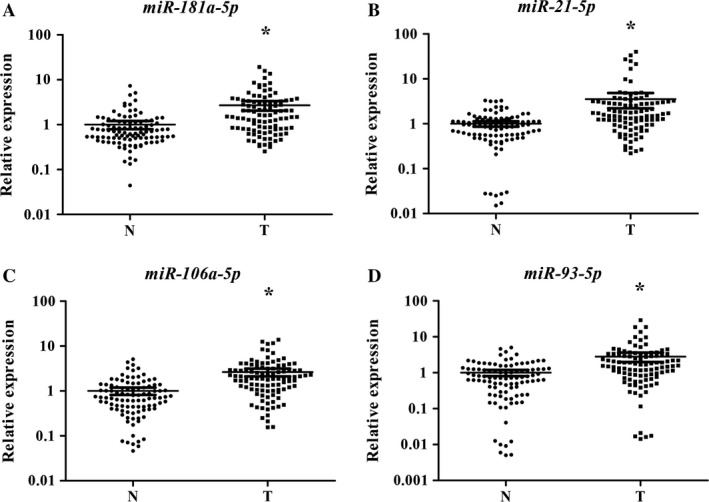
Expression levels of the four miRNAs in the plasma of 102 male LSCC patients and 101 NCs. (A) miR‐181a‐5p; (B) miR‐21‐5p; (C) miR‐106a‐5p; miR‐93‐5p; N, normal controls; T, tumor. Horizontal line: mean with 95% CI. *P < 0.001.

Twenty‐seven candidate miRNAs were tested by qRT‐PCR in training stage including 32 LSCC patients and 31 NCs. After that, a total of 12 significantly expressed miRNAs were demonstrated and were subsequently validated in the testing stage including 55 LSCC patients and 55 NCs (Table S1). Next, of those 12 miRNAs, consistent up‐regulation of four miRNAs (miR‐181a‐5p, miR‐21‐5p, miR‐106a‐5p, and miR‐93‐5p) were detected in the plasma samples of all LSCC patients, with mean fold change (FC) >1.5 and *P*‐value <0.05. Then, for validation purposes, all the four miRNAs identified were assessed to further validation stage with a cohort of 15 LSCC patients and 15 NCs. Consequently, we found that the expression trend of four miRNAs was concordant in those in the former three stages. Additionally, when the three stages were combined, all the four miRNAs presented significantly high expression in plasma of LSCC patients (Fig. 2). Consequently, a four‐miRNA signature was identified as a potential detection biomarker for LSCC patients.

### Diagnostic value of the four plasma miRNAs

**Figure 3:**
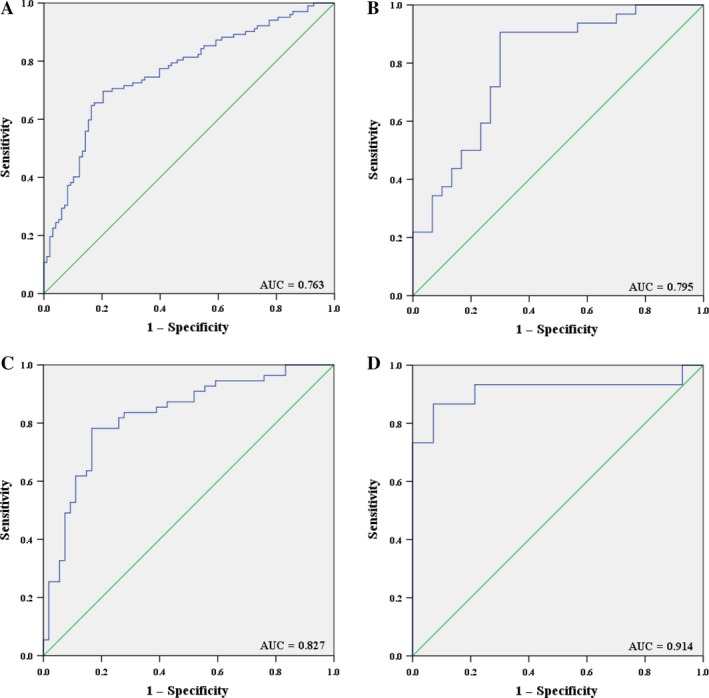
Receiver operating characteristic (ROC) curves for the four‐miRNA panel to discriminate LSCC patients from NCs. (A) The combined three phases of training, testing, and external validation phases (102 LSCC vs. 101 NCs); (B) training phase (32 LSCC vs. 31 NCs); (C) testing phase (55 LSCC vs. 55 NCs); (D) external validation (15 LSCC vs. 15 NCs). AUC: areas under the curve.

We conducted ROC curve analyses on each of the individual four plasma miRNAs to assess the diagnostic value for discriminating between LSCC patients and NCs in combined three stages (training stage, testing stage, and external validation stage). The AUCs for miR‐181a‐5p, miR‐21‐5p, miR‐106a‐5p, and miR‐93‐5p were 0.731 (95% confidence interval (CI): 0.661–0.800), 0.739 (95% CI: 0.670–0.808), 0.737 (95% CI: 0.667–0.807), and 0.687 (95% CI: 0.614–0.761), respectively (Fig. S1). As shown in Fig. 3A, we performed a risk score analysis on the combined cohorts and found that the four‐miRNA panel had greater abilities of separating capacity than one particular miRNA, evidenced by an AUC of 0.763 (95% CI: 0.696–0.829). Meanwhile, in the training, testing, and external validation stages, diagnostic capability of the four‐miRNA panel was also assessed separately. The AUCs for the panel were 0.795 (95% CI: 0.682–0.908; Fig. 3B), 0.827 (95% CI: 0.747–0.906; Fig. 3C), and 0.914 (95% CI: 0.791–1.000; Fig. 3D), respectively.

We further evaluated the relationship between the four miRNAs in plasma and tumor stage in all 101 LSCC patients. The corresponding AUCs of TNM stages I, II, and III were 0.785, 0.786, and 0.784, respectively (Fig. S2).

Meanwhile, four miRNAs were evaluated in patients with early stage (I + II) compared to the patients with advanced stage (III), but none of them showed different expression (Fig. S3).

### Identification of miRNA expression in tissues

**Figure 4:**
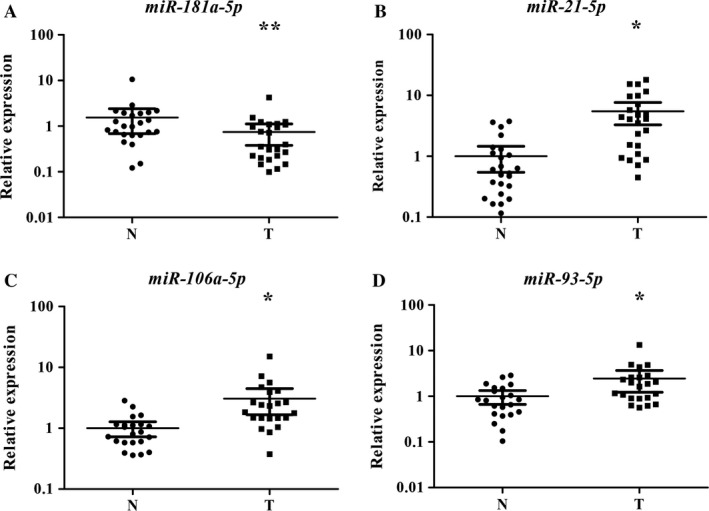
Expression of the four miRNAs in the tumor tissues of 23 pairs of LSCC patients. (A) miR‐181a‐5p; (B) miR‐21‐5p; (C) miR‐106a‐5p; miR‐93‐5p; N, normal controls; T, tumor. Horizontal line: mean with 95% CI. *P < 0.001.

Then, to explore the consistency of the four miRNAs in plasma and tissue of LSCC patients, their expression was detected in 23 pairs of tissue specimens of LSCC. Three miRNAs (miR‐21‐5p, miR‐106a‐5p, and miR‐93‐5p) had statistically higher expression level in tumor specimens than in normal tissues as is demonstrated in Fig. 4. However, contrary result showed that miR‐181a‐5p expressed statistically lower level in tumor samples. Additionally, the miRNA profiles of 32 male LSCC tissues and matched normal tissues from the TCGA database were assessed. But three of the four miRNAs (miR‐21‐5p, miR‐106a‐5p, and miR‐93‐5p) showed no significant result expect miR‐181a‐5p, which was found down‐regulated in tumor tissues as well (Fig. S4).

### Identification of miRNA expression in plasma exosomes

**Figure 5:**
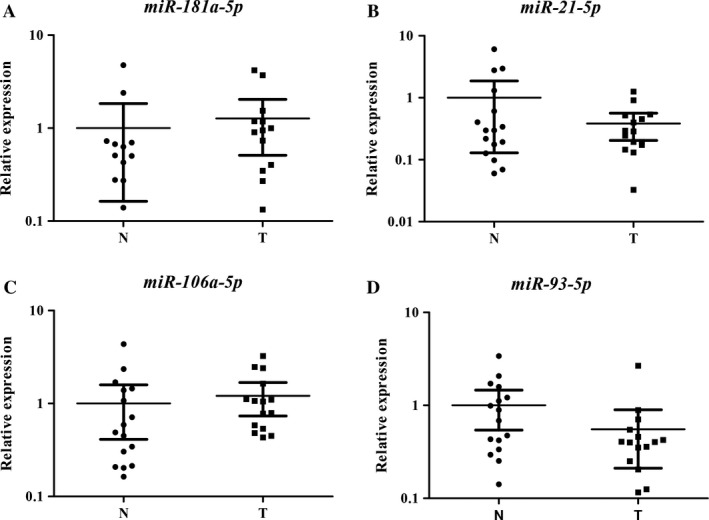
Expression of the four miRNAs in the plasma exosomes of 16 LSCC and 16 NCs. N, normal controls; T, tumor. *P < 0.001.

To assess the potential expression form of miRNAs in plasma, we examined exosomal miRNA expression level of the four identified miRNAs in 16 LSCC and 16 NC plasma samples. However, the results were not statistically significant in our study (Fig. 5). The p‐value for miR‐181a‐5p, miR‐21‐5p, and miR‐106a‐5p was 0.2701, 0.7624, and 0.1627, respectively. Although no result achieved statistical significance potentially due to the small sample size, interestingly, miR‐93‐5p in plasma exosomes showed low expression with borderline significance (*P* = 0.0544).

### Exploration of the four‐miRNA expression in female patient plasma

To further validate the diagnostic value of the miRNA panel in female patients, the expression levels of the four miRNAs were performed in three female patients with LSCC and five female NCs. As a result, except miR‐21‐5p (*P* = 0.0012), miR‐181a‐5p, miR‐106a‐5p, and miR‐93‐5p have no significant difference between female patients and NCs (Fig. S5).

### Bioinformatics analysis of candidate miRNAs

To decipher the potential function of the four identified miRNAs, DIANA‐TarBase v 7.0 was applied to screen the target genes of each miRNA. We put the four identified miRNAs into the DIANA‐miRPath v3.0, a pathway analysis Web server for miRNA targets to investigate the pathways with Kyoto Encyclopedia of Genes and Genomes (KEGG) analysis and Gene Ontology (GO) analysis (Table S2). KEGG analysis showed that several tumor‐related signaling pathways seemed to be regulated by the four miRNAs, such as FoxO signaling pathway, Hippo signaling pathway, and Prolactin signaling pathway. The pathways interaction of the four miRNAs also showed their tight relationship with Proteoglycans in cancer, Lysine degradation, and NSCLC. GO analysis demonstrated various signaling pathways in common as well.

## Discussion

NSCLC accounts for approximately 85% of all lung cancer, and LSCC is one of the major histological subtypes 2. miRNAs contribute to tumorigenesis and development of multifarious malignancies, which might be a treasure for cancer diagnosis and therapy, including lung cancer 15. However, few studies have characterized the profiles of miRNAs in LSCC patients.

In present study, we focused on LSCC in male patients and carefully established a procedure to identify a plasma miRNA signature for the disease. Compared to TaqMan platform, Exiqon miRNA qPCR panels have been proved more linear and sensitive in measuring miRNAs with relatively low abundance and utilized to conduct plasma miRNA profile in initial screening set 16. To control the false‐positive rate, our study performed three stages for validation (the training stage, the testing stage, and the external validation stage) using qRT‐PCR subsequently. Consequently, four up‐regulated plasma miRNAs (miR‐181a‐5p, miR‐21‐5p, miR‐106a‐5p, and miR‐93‐5p) showed higher accuracy and were identified for LSCC screening. It has been proved that complex patterns of miRNAs can offer more robust information on disease status than single miRNA. We daringly hypothesized that the four‐miRNA signature might become the candidate for noninvasive LSCC diagnosis in male Chinese patients.

To date, several researches have reported different profiles of miRNA expression in NSCLC, and all of the four miRNAs (miR‐181a‐5p, miR‐21‐5p, miR‐106a‐5p, and miR‐93‐5p) acquired in our study have been reported separately be valuable in NSCLC diagnosis 9, 17, 18. However, there has been a growing need for individualized treatment with efficacy and safety because of growing understanding of histologic and molecular differences among diverse subtypes of NSCLC. In addition, the majority of the studies concentrate on tissue samples deriving from surgical section or biopsy, which limits their application in LSCC detection. Liquid biopsy, with its noninvasive capability which allows repeated monitoring for broader molecular understanding of tumor, has become a prominent research topic in precision medicine for cancer. Hence, there is a great application prospect of circulating miRNA as to the diagnosis of LSCC.

Evidences indicate that the overexpression of miR‐106a‐5p could exert modulatory influences on VEGF‐A and Hif1a, two key factors in neovascularization 19. Xie et al. 17 suggested that miR‐106a‐5p could enhance NSCLC cell proliferation and metastasis by directly targeting PTEN, while a previous study showed the PTEN/PI3K/pAkt pathway involved in LSCC carcinogenesis 20. What is more, it was revealed that miR‐106a‐5p might act as an anti‐apoptotic factor and could confer the cisplatin resistance of NSCLC cells by targeting ABCA1 21. Circulating miR‐106a‐5p has been proved to show high levels in a wide range of other cancers, such as melanoma 22, colorectal cancer 23, gastric cancer 24, as well as esophageal squamous cell carcinoma 25. MiR‐93‐5p, as a miRNA in the miR‐106b~25 cluster and a paralog of the miR‐17~92 cluster, has been previously verified up‐regulated in NSCLC tissues 18, 26. Li et al. confirmed that up‐regulated miR‐93‐5p facilitated tumorigenesis and metastasis of NSCLC by activating oncogenic PI3K/Akt pathway through inhibition of LKB1, PTEN, and p21 expression 25. Meanwhile, miR‐93‐5p could enhance tumor angiogenesis of NSCLC by suppressing *β*‐TRCP2 27. Other target genes of miR‐93‐5p include large tumor suppressor homology 2 (LATS2) 28 and disabled homolog 2 (DAB2) 29, contributing to lung tumorigenesis. Additionally, up‐regulation of circulating miR‐93‐5p could also aid in the detection of acute myeloid leukemia 30, hepatocellular carcinoma 31, and nasopharyngeal carcinoma 32. MiR‐21‐5p as a well‐known onco‐miRNA was comprehensively discussed among a variety of malignancies, including NSCLC. Mature miR‐21‐5p was discovered significantly up‐regulated in blood, tissue, and sputum samples of NSCLC and was regarded as an independent negative prognostic factor for overall survival of NSCLC 8, 9, 10, 11, 12. Aberrantly increased miR‐21‐5p could promote lung tumorigenesis and inhibit cell apoptosis via the Ras/MEK/ERK and activate EGFR signaling pathway 33, 34. Besides, Xu et al. 35 claimed that miR‐21‐5p mediated cellular proliferation, invasion, migration, and apoptosis by targeting PTEN, RECK, and Bcl‐2 in LSCC. At present, a robust association of miR‐21‐5p expression in tumors with diagnostic effect was also observed in numerous solid tumors, such as colorectal cancer 36, gastric cancer 37, breast cancer 38, pancreatic cancer 39, and so on. Moreover, overexpression of miR‐21‐5p was correlated with poor survival of the patients with pancreatic cancer 40 and gastric cancer 41. Unlike the deregulation of the three miRNAs above acting in one direction consistently in a variety of cancers, miR‐181a‐5p was found up‐regulated in gastric cancer 42 and hepatocellular carcinoma 43, while down‐regulated in other carcinoma, such as glioblastomas 44, breast cancer 45, and lymphocytic leukemia 46. Furthermore, in Li's study, down‐regulation of miR‐181a‐5p could successfully sensitize cancer cells to chemotherapy probably by targeting PTEN 47, while Yang et al. 48 discovered overexpression of miR‐181a‐5p enhanced lymph node metastasis through regulating migration. These opposite results may ascribe to the different fundamental methods and various samples origin. According to our bioinformatics analysis, several signaling pathways were likely to be linked with the four‐miRNA panel. FoxO transcription factor acts as tumor suppressor in several malignancies, and by targeting the PI3K/Akt/FoxO signaling pathway, prostate cancer progression would be effectively suppressed in part 49. The Hippo pathway can regulate organ size in diverse species, whereas deregulation of the pathway may induce tumors and occur in a broad range of carcinomas, including lung cancer 50. Prolactin pathway can mediate diverse activities under the conditions of normal or abnormal, including malignancy 51. Of course, mechanisms between these signaling pathways and the four miRNAs are warranted to be delved into.

Among the four miRNAs, miR‐106a‐5p and miR‐93‐5p have been proved consistently overexpressed in serum of male LSCC patients in our former study, which further confirmed the diagnostic power of the two miRNAs in LSCC 14. It has been reported that blood clotting prerequisite to serum preparation leads to changes in the distribution of certain miRNAs, which might explain the differences in miRNA expression between serum and plasma samples 52. Meanwhile, Cao with his coworkers have discovered the up‐regulation of miR‐21 and miR‐93 in 54 pairs of LSCC samples and matched adjacent normal samples. The similar results proved the value of miR‐21 and miR‐93 in LSCC detection 53. Which can explain the difference is the discrepancy in processing tissue specimens, we utilized the FFPE samples while Cao took advantage of fresh‐frozen surgical specimens. Ayaz et al. 54 found that miR‐21‐5p and miR‐106a‐5p were expressed only in plasma of LSCC patients, while miR‐93‐5p was up‐regulated in LSCC compared to control group. It is not strange to have inconsistence given the reality that there were only 20 LSCC western patients (two females and 18 males) in his research, while we enrolled 102 LSCC eastern patients. What is more, we explored the expression of the four miRNAs in three female LSCC patients and five female NCs. Consequently, miR‐21‐5p showed significant up‐regulation, while the other three miRNAs have no significant difference in female patients compared to the female controls. More female LSCC patients and comprehensive data are required to validate our findings.

To further strengthen the hypothesis we investigated, plasma miRNAs for LSCC detection were also explored in tissues. Intriguingly, three of the four miRNAs described in plasma showed consistently high expression in LSCC tissue samples expect miR‐181a‐5p. MiR‐181a‐5p was found totally differently expressed between tissue samples and plasma samples in LSCC. We inferred that miR‐21‐5p, miR‐106a‐5p, and miR‐93‐5p in LSCC plasma could be released from primary cancer cells for consistent expression tendency between plasma and tissue specimens. MiR‐181a‐5p was found down‐regulated in NSCLC tissues and significantly inhibited NSCLC by targeting oncogene KRAS in Ma's study 55. We speculated that circulating miR‐181a‐5p might be poured forth from the normal tissue to suppress the cell proliferation. In addition, by analyzing the miRNA profiles of male LSCC patients based on TCGA dataset, the reduced expression of miR‐181a‐5p was in accordance with our finding in tissue while the other three miRNAs showed no difference. We reasonably speculated that there existed discrepancy of the miRNA expression profiles between the Asian and Caucasian. Differences in gene mutation rates between different ethnic groups have been verified in previous research 56. Anyway, it is necessary to validate the findings in more LSCC tissue samples.

Stability mechanisms of circulating miRNAs are still elusive. Therefore, to better explore potential form of miRNAs in plasma, expression of the four miRNAs was also tested in exosomes. Exosomes are membrane‐enclosed extracellular vesicles with a diameter of 40~100 nm. Almost all types of cells can secrete exosomes under conditions whether normal or stressful, particularly cancer cell 57. These vesicles carry plenty of biological molecules, such as RNAs, DNAs, proteins, as well as lipids, and protect these signaling molecule away from enzymes 58. It was reported that exosomal miRNAs could participate in tumor metastasis 59. Grange et al. 60 put forward that exosomes might play a noticeable role in triggering angiogenesis, promoting formatting a premetastatic niche and enhancing formation of lung metastasis. However, in our study, none of exosomal miR‐181a‐5p, miR‐21‐5p, and miR‐106a‐5p showed different expression levels in peripheral plasma between LSCC patients and NCs, potentially owing to the small sample case. Nevertheless, miR‐93‐5p in plasma exosomes showed low expression with borderline significance (*P* = 0.0544). We assumed that the majority of miR‐93‐5p might bind to proteins in plasma of LSCC patients, such as the Ago2 ribonucleoprotein complex and high‐density lipoprotein 52. Further studies are warranted to be explored and validated the phenomena and mechanisms.

Although our study has identified four plasma miRNAs for LSCC detection, there still exist some limitations. Firstly, the four‐miRNA signature was identified for LSCC patients with stage I, II, or III as patients all underwent surgical operation. But diagnostic value for patients with metastasis could not be evaluated. Thus, future studies with advanced stage LSCC samples should be performed. Secondly, the precise mechanisms by which these four miRNAs facilitate NSCLC progression remain largely obscure. Further researches in vitro and in vivo will enrich the knowledge of this nascent field necessarily. Thirdly, the sample size in our study is relatively small and studies on larger cohorts to confirm our data are required.

## Conclusion

In summary, we established a four‐miRNA signature (miR‐181a‐5p, miR‐21‐5p, miR‐106a‐5p, and miR‐93‐5p) in plasma associated with male LSCC patients. This pattern of plasma miRNAs could potentially pose as a noninvasive and efficiency biomarker for LSCC diagnosis. Larger cohorts of participants are warranted to enroll to explore the clinical application and mechanisms of four plasma miRNAs.

## Conflict of Interest

None declared.

## Supporting information


**Figure S1.** ROC curve analyses of each miRNA to discriminate LSCC patients from NCs in the combined three phases.
**Figure S2.** ROC curves for the ability of the four‐miRNA panel to differentiate LSCC patients with different TNM stages.
**Figure S3.** Expression level for the ability of the four miRNAs to differentiate LSCC patients with stage I+II and patients with stage III.
**Figure S4.** Expression levels of the four miRNAs the tumor tissues of 32 pairs of male LSCC patients in TCGA data.
**Figure S5.** Expression levels of the four miRNAs in the plasma of 3 female LSCC patients and 5 female NCs.
**Table S1.** Differently expressed miRNAs in the screening phase.
**Table S3.** Expression levels of the three miRNAs in the peripheral serum in the training and testing stages (presented as mean ± SD).

**Table S2.** DIANA‐miRPath v3.0 analysis for the target genes of each candidate miRNAs identified from DIANA‐TarBase v7.0 database to decipher the potential function of the above miRNAs.
