# Supplementary material for: Identification of four plasma microRNAs as potential biomarkers in the diagnosis of male lung squamous cell carcinoma patients in China
Source: Cancer Med. 2018 Apr 19;7(6):2370–81. doi: 10.1002/cam4.1490 (PMC6010830; doi:10.1002/cam4.1490)
Supplement: Supplementary file 1 — Figure S1. ROC curve analyses of each miRNA to discriminate LSCC patients from NCs in the combined three phases. Figure S2. ROC curves for the ability of the four‐miRNA panel to differentiate LSCC patients with different TNM stages. Figure S3. Expression level for the ability of the four miRNAs to differentiate LSCC patients with stage I+II and patients with stage III. Figure S4. Expression levels of the four miRNAs the tumor tissues of 32 pairs of male LSCC patients in TCGA data. Figure S5. Expression levels of the four miRNAs in the plasma of 3 female LSCC patients and 5 female NCs. Table S1. Differently expressed miRNAs in the screening phase. Table S3. Expression levels of the three miRNAs in the peripheral serum in the training and testing stages (presented as mean ± SD). [file CAM4-7-2370-s001.docx]

**Figure S1** ROC curve analyses of each miRNA to discriminate LSCC patients from NCs in the combined three phases. a: miR-181a-5p; b: miR-21-5p; c: miR-106a-5p; miR-93-5p.


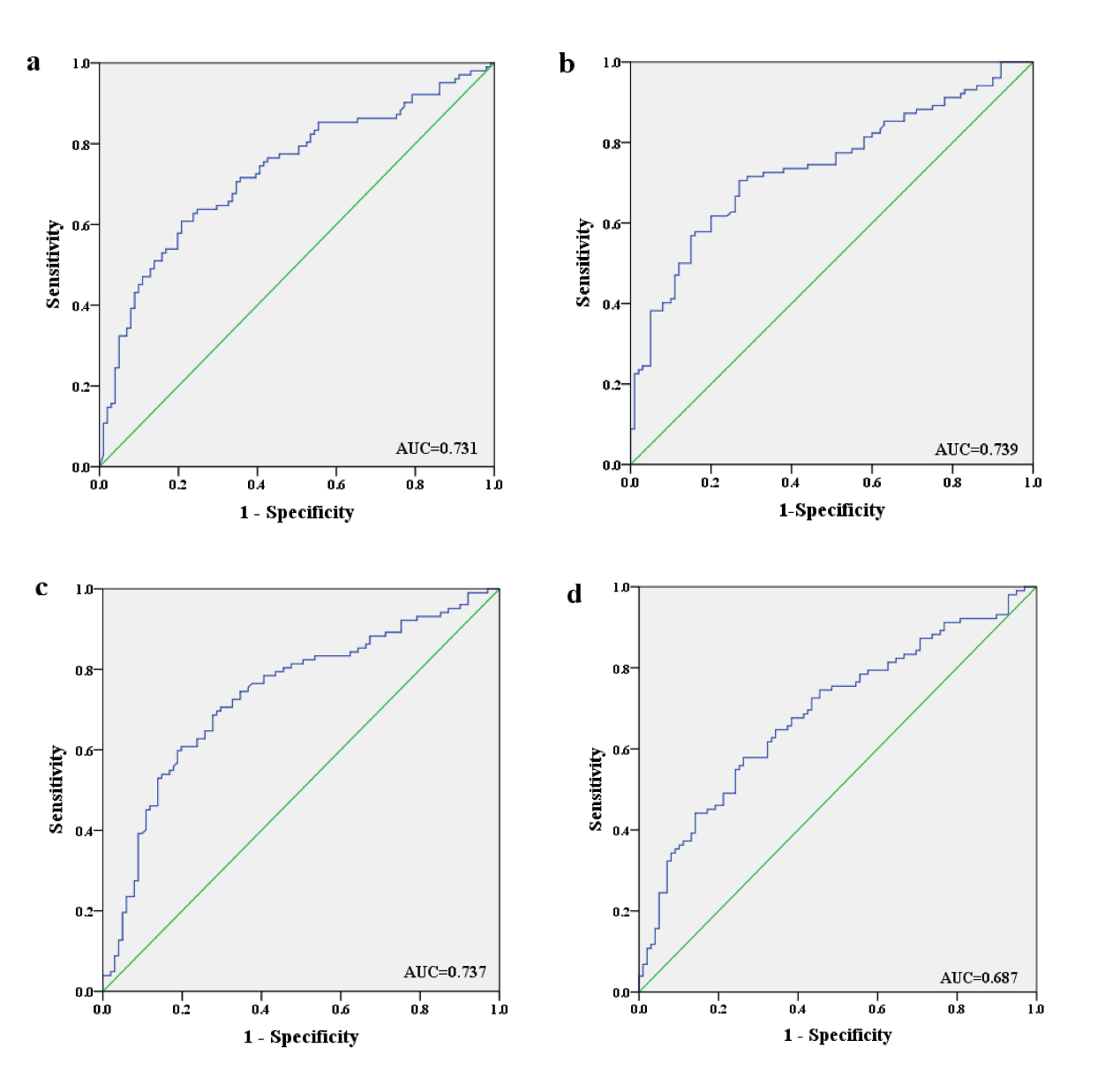


**Figure S2** ROC curves for the ability of the four-miRNA panel to differentiate LSCC patients with different TNM stages. a: stages I, b: stage II, c: stage III.


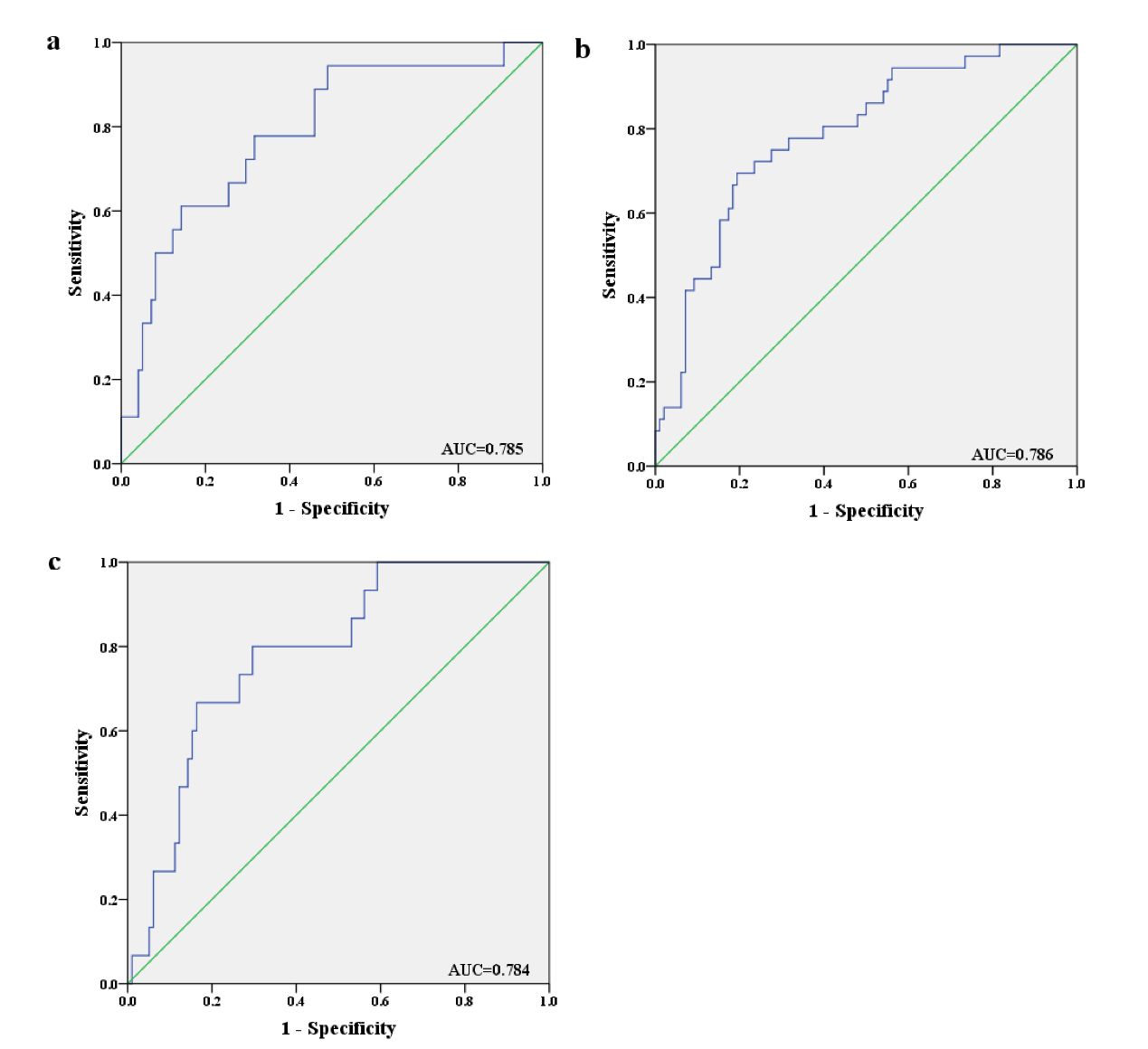


**Figure S3.** Expression level for the ability of the four miRNAs to differentiate LSCC patients with stage I+II and patients with stage III. LSCC: lung squamous cell carcinoma


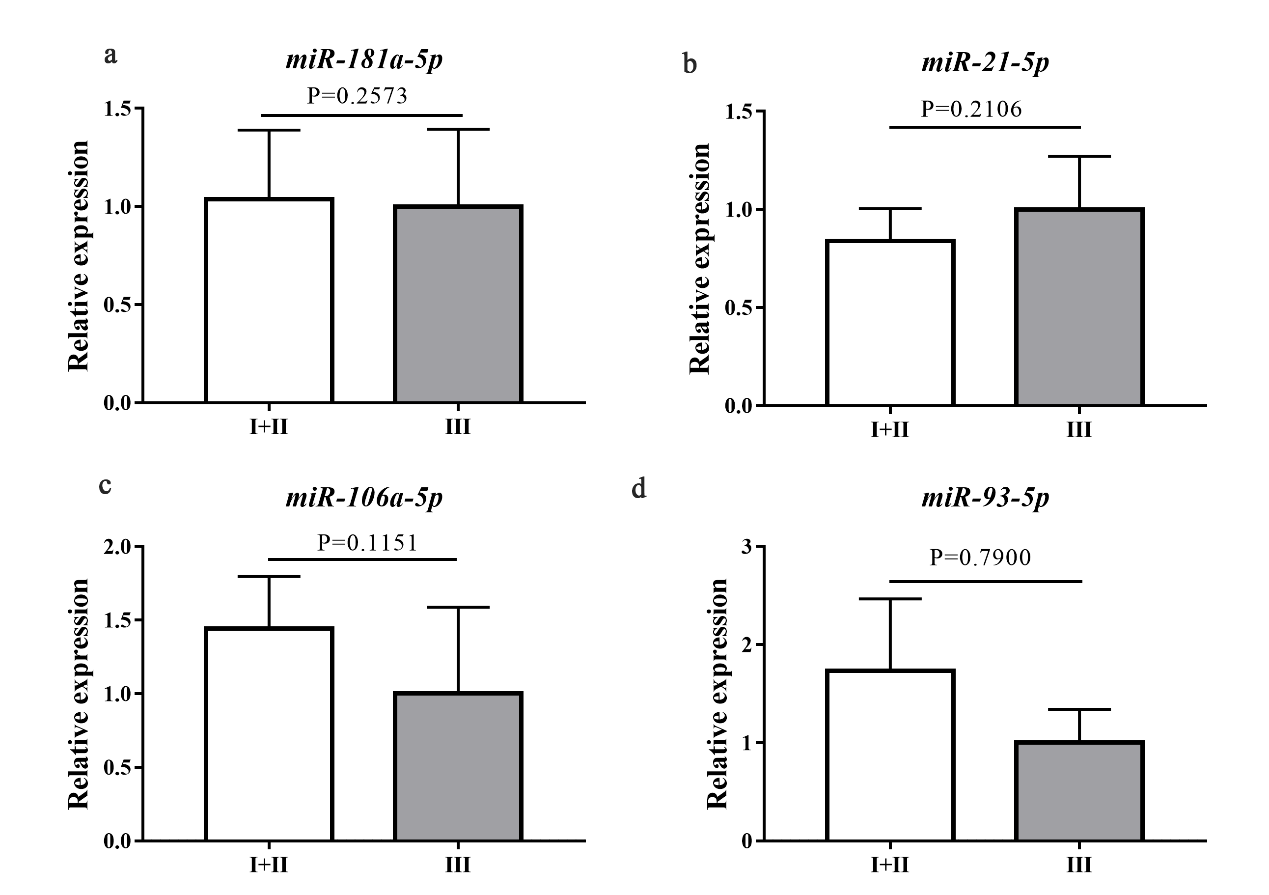


**Figure S4** Expression levels of the four miRNAs the tumor tissues of 32 pairs of male LSCC patients in TCGA data. a: miR-181a-5p; b: miR-21-5p; c: miR-106a-5p; miR-93-5p; N: normal controls; T: tumor. * : *P < 0.001*.


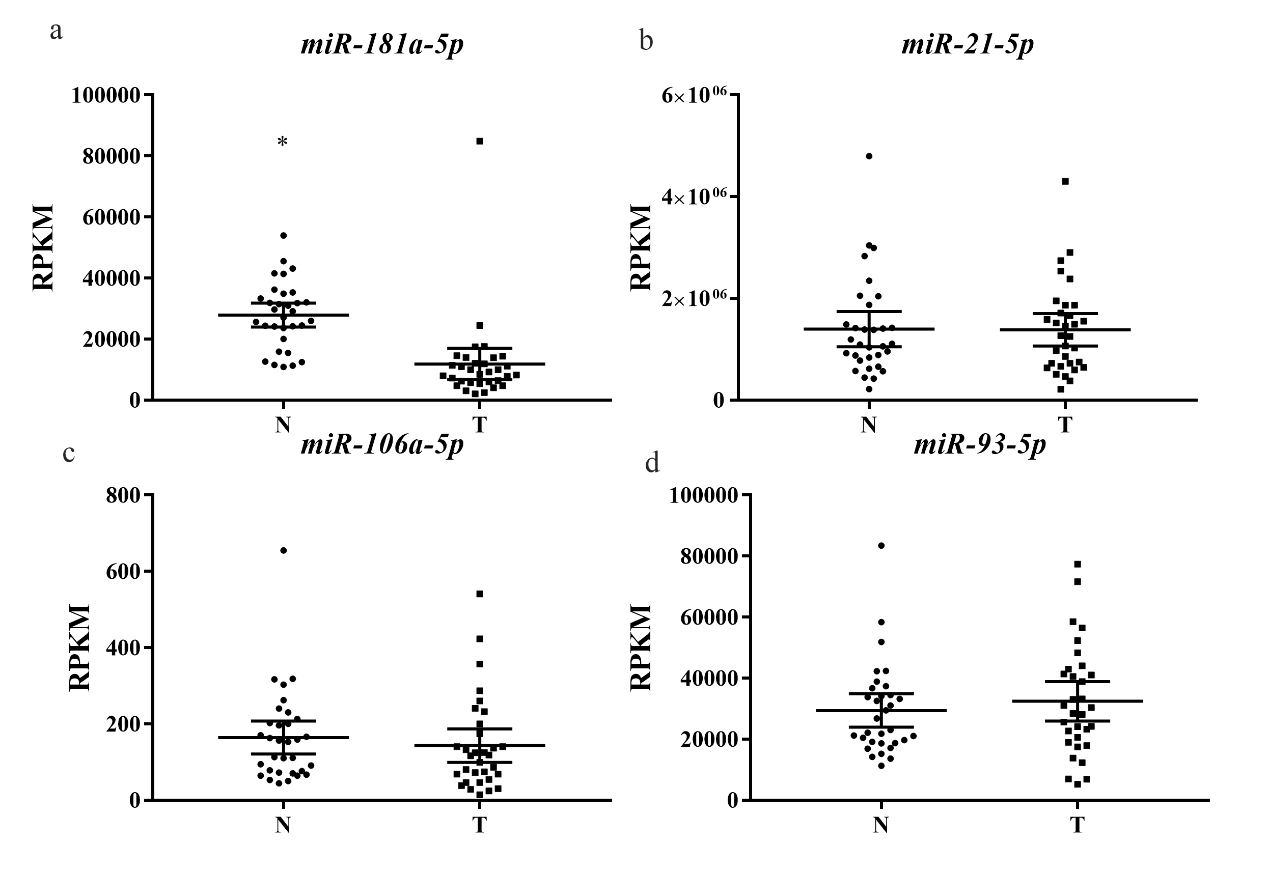


**Figure S5** Expression levels of the four miRNAs in the plasma of 3 female LSCC patients and 5 female NCs. a: miR-181a-5p; b: miR-21-5p; c: miR-106a-5p; miR-93-5p; N: normal controls; T: tumor. * : *P < 0.001*.


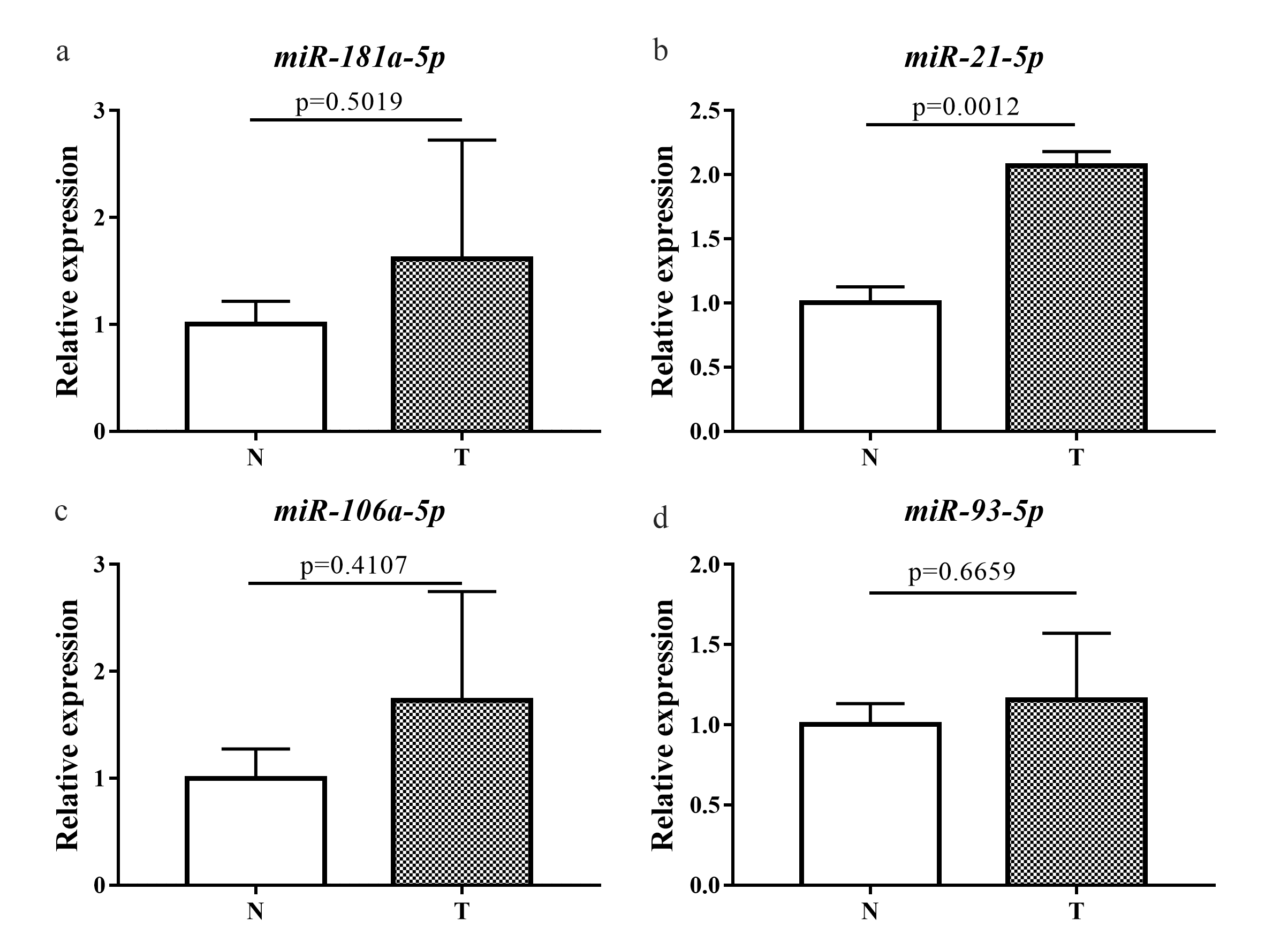


| **Table S1: Differently expressed miRNAs in the screening phase** | | | | | | | | | | |
| --- | --- | --- | --- | --- | --- | --- | --- | --- | --- | --- |
| **miRNA** | | **FC (Exiqon panels)** | | | | | | | | |
|  |  | **pool1** | | **pool2** | **pool3** | | | **Mean fold** | | |
| hsa-miR-106a-5p | | 2.046685835 | | 1.713745942 | 1.660504923 | | | 1.8069789 | | |
| hsa-miR-133a-3p | | -4.220008778 | | -1.61896635 | -2.422216802 | | | -2.753730643 | | |
| hsa-miR-145-5p | | -4.981374925 | | -3.571648237 | -1.585162782 | | | -3.379395315 | | |
| hsa-miR-181a-5p | | 2.340451712 | | 2.939726624 | 1.877241163 | | | 2.3858065 | | |
| hsa-miR-19a-3p | | 2.078668933 | | 4.358502388 | 2.867197729 | | | 3.10145635 | | |
| hsa-miR-200a-3p | | 5.710800553 | | 6.243785606 | 9.619720652 | | | 7.191435603 | | |
| hsa-miR-21-5p | | 1.537594414 | | 1.762581539 | 1.814508733 | | | 1.704894895 | | |
| hsa-miR-30b-5p | | -2.015687515 | | -2.308858885 | -3.126143491 | | | -2.483563297 | | |
| hsa-miR-424-5p | | 2.118571765 | | 2.127916072 | 1.951136214 | | | 2.065874684 | | |
| hsa-miR-497-5p | | 1.767128559 | | 8.431856124 | 1.534434646 | | | 3.911139776 | | |
| hsa-miR-590-5p | | -2.070659018 | | -2.205371956 | -2.090830341 | | | -2.122287105 | | |
| hsa-miR-199a-3p | | 3.942381524 | | 6.866041925 | 5.992917444 | | | 5.600446964 | | |
| hsa-miR-133b | | -3.257244461 | | -7.210218601 | -3.423146413 | | | -4.630203158 | | |
| hsa-miR-143-3p | | -6.948949999 | | -5.135465306 | -1.762006199 | | | -4.615473835 | | |
| hsa-miR-15b-3p | | 5.755313037 | | 3.316519481 | 8.748802134 | | | 5.940211551 | | |
| hsa-miR-199a-5p | | 5.518028852 | | 1.758079122 | 3.315855183 | | | 3.530654386 | | |
| hsa-miR-19b-3p | | 1.681548045 | | 1.597466857 | 1.735463773 | | | 1.671492892 | | |
| hsa-miR-210-3p | | 2.205829069 | | 2.276554899 | 9.174214675 | | | 4.552199548 | | |
| hsa-miR-29b-2-5p | | 2.434863675 | | 2.059950971 | 7.272385112 | | | 3.922399919 | | |
| hsa-miR-382-5p | | 5.398265748 | | 5.302155567 | 2.359120319 | | | 4.353180544 | | |
| hsa-miR-326 | | -8.597385517 | | -2.490267979 | -1.982371017 | | | -4.356674838 | | |
| hsa-miR-425-5p | | 1.513393339 | | 1.871139992 | 2.12222703 | | | 1.835586787 | | |
| hsa-miR-532-3p | | -1.794821038 | | -2.162327431 | -2.3249086 | | | -2.094019023 | | |
| hsa-miR-93-5p | | 1.781488289 | | 1.868738239 | 2.827388242 | | | 2.159204924 | | |
| hsa-miR-103-3p | | 1.911162565 | | 1.550982599 | 1.537449475 | | | 1.666531546 | | |
| hsa-miR-16-5p | | 1.765418229 | | 1.962880576 | 1.941892045 | | | 1.890063617 | | |
| hsa-miR-20b-5p | | 1.581905699 | | 2.67386013 | 2.797258147 | | | 2.351007992 | | |
| FC: fold change; | |  | |  |  | | |  | | |
| **Table S3: Expression levels of the three miRNAs in the peripheral serum in the training and testing stages (presented as mean ± SD). FC: fold change** | | | | | | | | | |  |
| **miRNA** | **Training stage** | | | | | | | |  |  |
|  | **Controls** | | **Cases** | | | **FC** | **P value** | |  |  |
| miR-106a-5p | 4.37±0.66 | | 6.17±0.84 | | | 3.48 | P=0.000 | |  |  |
| miR-133a-3p | 4.75±0.86 | | 6.11±0.95 | | | 2.58 | P=0.004 | |  |  |
| miR-181a-5p | 7.17±1.41 | | 8.77±0.69 | | | 3.04 | P=0.006 | |  |  |
| miR-19a-3p | 5.37±0.85 | | 6.22±0.67 | | | 1.79 | P=0.014 | |  |  |
| miR-21-5p | 3.17±0.94 | | 4.21±0.56 | | | 2.06 | P=0.02 | |  |  |
| miR-30b-5p | 6.84±0.90 | | 7.90±0.89 | | | 2.08 | P=0.012 | |  |  |
| miR-590-5p | 10.34±0.65 | | 11.32±0.77 | | | 1.98 | P=0.009 | |  |  |
| miR-93-5p | 2.55±0.55 | | 4.17±0.80 | | | 3.08 | P=0.000 | |  |  |
| miR-451a | 2.38±0.81 | | 4.09±0.87 | | | 3.25 | P=0.000 | |  |  |
| miR-199a-5p | 9.60±1.26 | | 11.37±0.82 | | | 3.41 | P=0.001 | |  |  |
| miR-19b-3p | 2.40±0.79 | | 3.49±0.85 | | | 2.13 | P=0.01 | |  |  |
| miR-425-5p | 6.46±0.85 | | 7.81±0.84 | | | 2.56 | P=0.002 | |  |  |
| miR-532-3p | 10.31±1.06 | | 11.42±1.16 | | | 2.17 | P=0.028 | |  |  |
